# A base composition analysis of natural patterns for the preprocessing of metagenome sequences

**DOI:** 10.1186/1471-2105-14-S11-S5

**Published:** 2013-11-04

**Authors:** Oliver Bonham-Carter, Hesham Ali, Dhundy Bastola

**Affiliations:** 1 College of Information Science & Technology School of Interdisciplinary Informatics Peter Kiewit Institute University of Nebraska at Omaha, Omaha, NE, USA

## Abstract

**Background:**

On the pretext that sequence reads and contigs often exhibit the same kinds of base usage that is also observed in the sequences from which they are derived, we offer a base composition analysis tool. Our tool uses these natural patterns to determine relatedness across sequence data. We introduce spectrum sets (sets of motifs) which are permutations of bacterial restriction sites and the base composition analysis framework to measure their proportional content in sequence data. We suggest that this framework will increase the efficiency during the pre-processing stages of metagenome sequencing and assembly projects.

**Results:**

Our method is able to differentiate organisms and their reads or contigs. The framework shows how to successfully determine the relatedness between these reads or contigs by comparison of base composition. In particular, we show that two types of organismal-sequence data are fundamentally different by analyzing their spectrum set motif proportions (coverage). By the application of one of the four possible spectrum sets, encompassing all known restriction sites, we provide the evidence to claim that each set has a different ability to differentiate sequence data. Furthermore, we show that the spectrum set selection having relevance to one organism, but not to the others of the data set, will greatly improve performance of sequence differentiation even if the fragment size of the read, contig or sequence is not lengthy.

**Conclusions:**

We show the proof of concept of our method by its application to ten trials of two or three freshly selected sequence fragments (reads and contigs) for each experiment across the six organisms of our set. Here we describe a novel and computationally effective pre-processing step for metagenome sequencing and assembly tasks. Furthermore, our base composition method has applications in phylogeny where it can be used to infer evolutionary distances between organisms based on the notion that related organisms often have much conserved code.

## Introduction and related work

During a DNA sequencing task, the nucleotides of the reads or contigs must be placed in the correct order to reconstruct the original sequence. This sequencing task is particularly challenging when working with a metagenomic task, which requires one to gather and order similar sequences from a number of different organisms. This metagenomic technique has been extensively discussed in [1,2] and a framework to infer phylogenetic relationships (patterns) among assemblages of microorganisms has been developed [3]. This approach is expected to help improve assembly projects by reducing search spaces when grouping related sequence fragments. Massively parallel *next-generation *sequencing technologies (a major technological rebirth of the former Sanger methods of the 1980's [4]) provide ultrahigh throughput results at a low cost but the reads are often too short to be able to determine their adjacency. In [5], the authors describe a novel method for *de novo *assembly of large genomes from short read sequences which they used to assemble two giant genomes: the Asian and African human genome sequences.

Some of the limitations encountered in the assembly process include read coverage and size. The absence of placement information such as read coverage forms a bottleneck in the reassembly process [6,7]. When the read sequences are very short, then special procedures must be taken to maximize their informational content to achieve placement evidence. For this work, it may be necessary to form contigs by de novo assembly methods as in [8]. Despite these limitations, technologies such as *Velvet *and *Oases *have been used for many genome assembly projects [9,10] and [11]. Assembling reads using approaches from probability theory, or from the memory-based, are gaining popularity. This was determined by Zhang *et. al*. [12] who compared the performance of eight distinct tools (i.e., SSAKE, VCAKE, QSRA, SHARCGS, Edena, Velvet, SOAPdenovo, and Taipan) against eight groups of simulated datasets.

**Figure 1:**
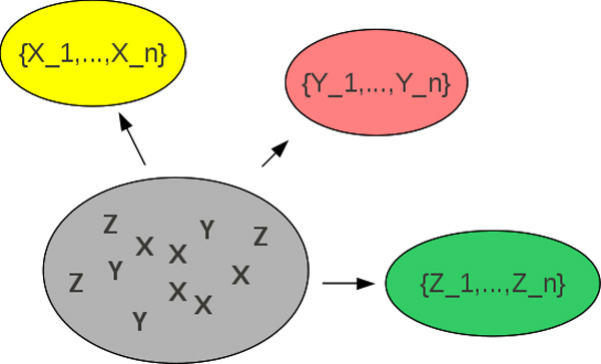
Sequence fragments are separated into groups (called,"bins") of relatedness by a quick pre-proccessing step. This graphic taken from our previous work in [33].

In metagenomic studies, where there are different kinds of reads or contigs mixed together into the same pool, the task of separating them back into *n*-distinct groups, becomes an NP-hard problem. Although a researcher may choose to determine their order using some computational tools, as described in Figure 1, this still is an NP-hard problem to separate the sequence data.

Furthermore, this difficulty of separating the reads may prevent the assembly tools from ever being used optimally. In [13], the authors discuss the problem of filtering the reads or contigs into smaller groups for better management. Time and productivity can be saved by these pre-processing steps where related sequence material is placed into a bin (here called, *binning*) to reduce search spaces for reconstructing entire sequences or genomes. It is therefore important to perform efficient binning steps to save costs in the sequencing task to reduce the work-load in an assembly project.

Chromosomal material across different genera were organized into species-specific groupings by virtue of the motif composition which was contained in the DNA [14]. In our study, we present a similar framework of organizing samples of DNA by their motif content. Our method differs from the authors' work, however, because it could be applied to smaller sequence fragments than chromosomes and it also employs motifs of similar base-composition to *associate *(e.g., bin) sequences of different organisms into related groups. Our set of motifs are biologically relevant since they were derived from known bacterial restriction sites. We permuted the base composition of the bases found in a particular restriction site to generate a list of all possible motifs of the same composition. Here, all the motifs belonging to a set of the same base composition is said to form a *spectrum set*. We show that an organism's recognition sequence belongs to only one of the four possible non-palindromic spectrum sets. Furthermore, each set must be strategically selected for successful sequence binning.

Our hypothesis is that a restriction site base composition algorithm can be used to separate and bin the sequence material from several different organisms. Our method compares the spectrum set motif proportions between sequences and uses this knowledge to separate them. For instance, if the motifs have similar proportions across two sequences, then there is evidence to suggest that the sequences are related to each other in some way. Here, this relation is called an association. In summary, our work stands apart from the traditional assembly pre-processing methods found in wet-labs since our method relies on statistics alone to find likenesses across sequence material to discover associations and bin the sequence data.

## Methods

### Genome sequences

The genomic DNA sequences were studied from six different phylogenetic groups (Actinobacteria, Firmicutes and Proteobacteria) shown in Table 1. The genomes and chromosomes for this study were downloaded from Genbank (a public and international online database) and were manipulated with tools which we describe below. All sample genomes were at least 1 Mb in length. There is evidence to suggest that GC-groups have a tendency to mutate to AT groups [15,16]. Furthermore, it is thought that similar GC composition implies similar genomic structure [17]. In light of this knowledge, our analysis was drawn from bacteria comprising many raised and lowered levels of GC-content.

**Table 1:** Genera for the study

Organism	Contig Originator	Division
*Bifidobacterium longum* *Mycobacterium bovis* *Clostridium tetani* *Staphylococcus aureus* *Burkholderia pseudomallei* *Campylobacter jejuni*	NC_004307 NC_002945 NC_004557 NC_007622 NC_012695 NC_008787	Actinobacteria Actinobacteria Firmicutes Firmicutes Proteobacteria Proteobacteria

This table displays the genera used in our study. The *Read Originator *column displays the sequences which we processed via MetaSim for its reads. To determine their general associative behaviors, we studied ten trials of five freshly drawn reads. We chose two organisms from each of the three divisions from which to draw our contigs.

In each experiment, ten trials of five freshly drawn reads were studied. We used MetaSim [18] to create artificial contigs (or reads) similar to those of an actual assembly project. Each set of contigs (or reads) was extracted from the *Contig Originator *organism of Table 1. We applied all four spectrum sets to determine the proportional distributions used for the leaf weights in our heatmap trees. We placed randomly selected contigs in each test. There were also several other related organisms added to each pool to test and further determine the association behaviors.

We found very similar trends in each division. We illustrate them by discussing the arbitrarily chosen the organisms *Staphylococcus *and *Clostridium *of the Firmicutes division. The results from the other divisions featured in Table 1, Proteobacteria and Actinobacteria, were very similar to the findings of the Firmicutes.

### Read and contig sequences

The synthetic data was made up of shorter reads of less than 1 kbp and were generated utilizing the *454 *framework that was offered by the MetaSim software tool. MetaSim selects its reads by a statistical approach according to user input. The software simulates the approaches of both Sanger sequencing and Roche's 454 (sequencing-by-synthesis). The maximum allowed length of contigs by MetaSim is 1 Kbp and so the longer reads or contigs for this study (1 Kbp 30 Kbp) had to be generated by our own tool, which also follows the 454 (sequencing-by-synthesis) method. We created longer reads or contigs of lengths 2 kbp, 5 kbp, 10 kbp and 30 kbp for an exhaustive study using this tool. Although it may appear that some of these reads are unnaturally long, we note that the typical lengths of reads appear to be growing as the sequencing technology improves and evolves.

In our experiments, we ran binning tests containing many reads or contigs but due to redundancy in the outcome of the analysis, our tests required only about five to ten reads or contigs to display the relevant trends. This small set of sequence data was acceptable to our work because we often observed that nearly all of the reads of a larger set had very similar distributions of motifs content from the spectrum sets.

### Motifs

REBASE [19] is an online database of information concerning bacterial restriction enzymes and their recognition sites. Each of the organisms (*Campylobacter*, *Burkholderia*, *Bifidobacterium*, *Mycobacterium*, *Clostridium*, *Staphylococcus*) were queried at REBASE for their organism-specific, palindromic recognition site sequences of length-6. This length was desirable for our work because (1) it is a common size in bacteria, mitochondria and plasmids and, (2) it is statistically interesting. For example, let *A *be the size of the DNA alphabet {A, C, G, T} (four elements) and let *L *be the motif length. There are $A^{\frac{L}{2}}=4^{\frac{6}{2}}=4^{3}=64$ possible palindromic sites available from the set of all possible length-6 words, *A^L ^*= 4^6 ^= 4096. When compared to the seemingly spontaneous occurrence rates of the shorter motifs, these longer words are less likely to be random occurrences along the genome.

#### Base compositions and spectrum sets

**Figure 2:**
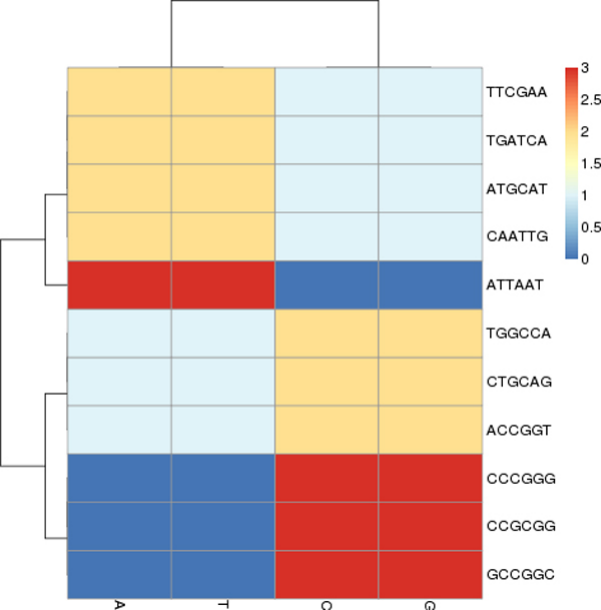
The spectrum set taken from the four restriction sites of the *Clostridium *genera. There are ten unique recognition sites covering all four spectrum sets (shown in Figure 4). This graphic taken from our previous work in [33].

**Figure 3:**
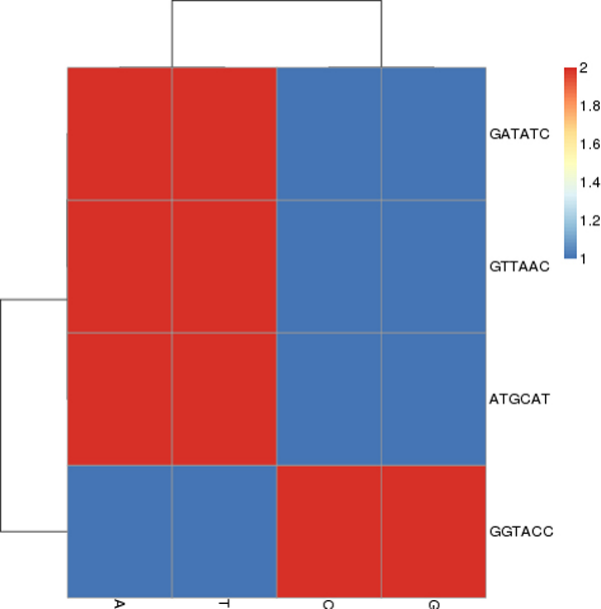
The spectrum set taken from the four restriction sites of the *Staphylococcus *Genera. The motif *ATGCAT *is common to *Clostridium*. This graphic taken from our previous work in [33].

**Figure 4:**
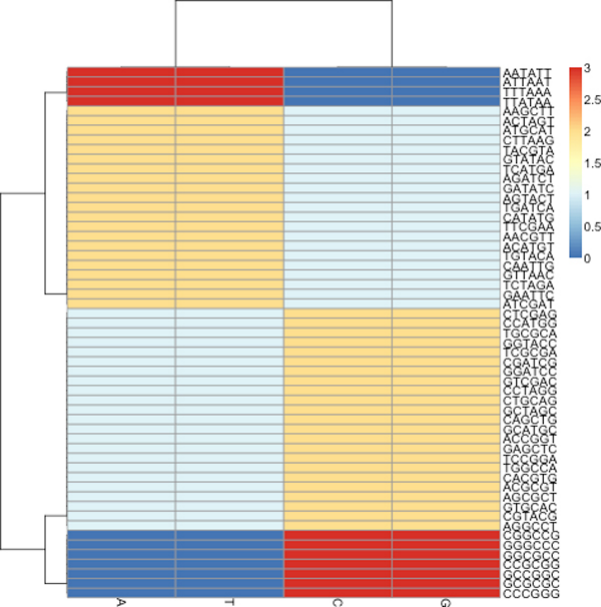
From its base composition, each bacterial restriction site fits into only one of the four spectrum sets, featured by unique color patterns. The motifs of each set are made up by the permutations of one of the following words; *AAATTT*, *AATTCG*, *CCGGAT *or *CCCGGG*. This result taken from our previous work in [33].

There are usually several uniquely spelled, palindromic recognition sequences of length-6 for each bacterial organism according to REBASE. For example, *Clostridium *has eleven recognition sequences (Figure 2), and *Staphylococcus *(Figure 3) has only four. It is typically rare to find common recognition sequences between two organisms however, in this case, *ATGCAT *is common to both. Consulting REBASE, we found all the known restriction sites and placed each into one of four unique sets according to their DNA compositions. In Figure 4, we show this grouping of all restriction sites. We call these sets, *spectrum sets *where each element of a set contains the same count of each base. We name each set by the following motifs: *AAATTT*, *AATTCG*, *CCGGAT *and *CCCGGG*. For example, the motifs, *ATTTAA*, *AATTTA*, *TAAATT *and *TTAAAT*, are all elements belonging to the *AAATTT-*spectrum set. A DNA word, *w *is palindromic if *w *== *reversed*[*Complemented*(*w*)]. We do not consider palindromes in our spectrum sets, although many of the restriction sites of restriction modification systems are naturally palindromic, since they are thought to be avoided in the genome [20]. This avoidance property may confuse our results since we are investigating their occurrences in a sequence. Table 2 lists the sizes of each set.

**Table 2:** Members of the spectrum sets

Set Seed	Available Motifs
AAATTT	12
CCCGGG	12
AATTCG	156
CCGGAT	156

The numbers of available motifs belonging to each spectrum. The motifs in the spectrum set are nonpalindromic and are permutations of the set seeds. The set created from the permutation of *AATTCG *is called, the *AATTCG-*spectrum, for example.

### Proportions

We use proportions, not frequencies, in our study of motif content because we are only using a subset of the set of all possible motifs of length-6. We ignored overlapping palindromes (no nested motifs) in the sequences for simplicity. The motif occurrence data in the sequences was normalized to make the comparisons meaningful. We determine the proportionality for each motif in a set across a genome by the following Equation 1:

The proportion of,

(1) $$m_{i}\;\text{in}\;S_{L}=\frac{count\left(m_{i}\right)*\left|m_{i}\right|}{\left|S_{L}\right|}$$

where *m_i _*is a motif, *S_L _*is a sequence fragment (a read, contig or genome), *count*(*m_i_*) represents the number of occurrences of *m_i _*found in *S_L_*, and *|m_i_| *and *|S_L_| *are the lengths of the motif and the sequence, respectively. For each motif in a spectrum set, the proportion of sequence that is made up of the motif is calculated by this equation. For each spectrum set, a vector is created from all proportions to be applied to a clustering analysis by hclust: a command in the R Statistical software [21]. The result of the analysis is a heatmap [22] to determine the associations.

**Figure 5:**
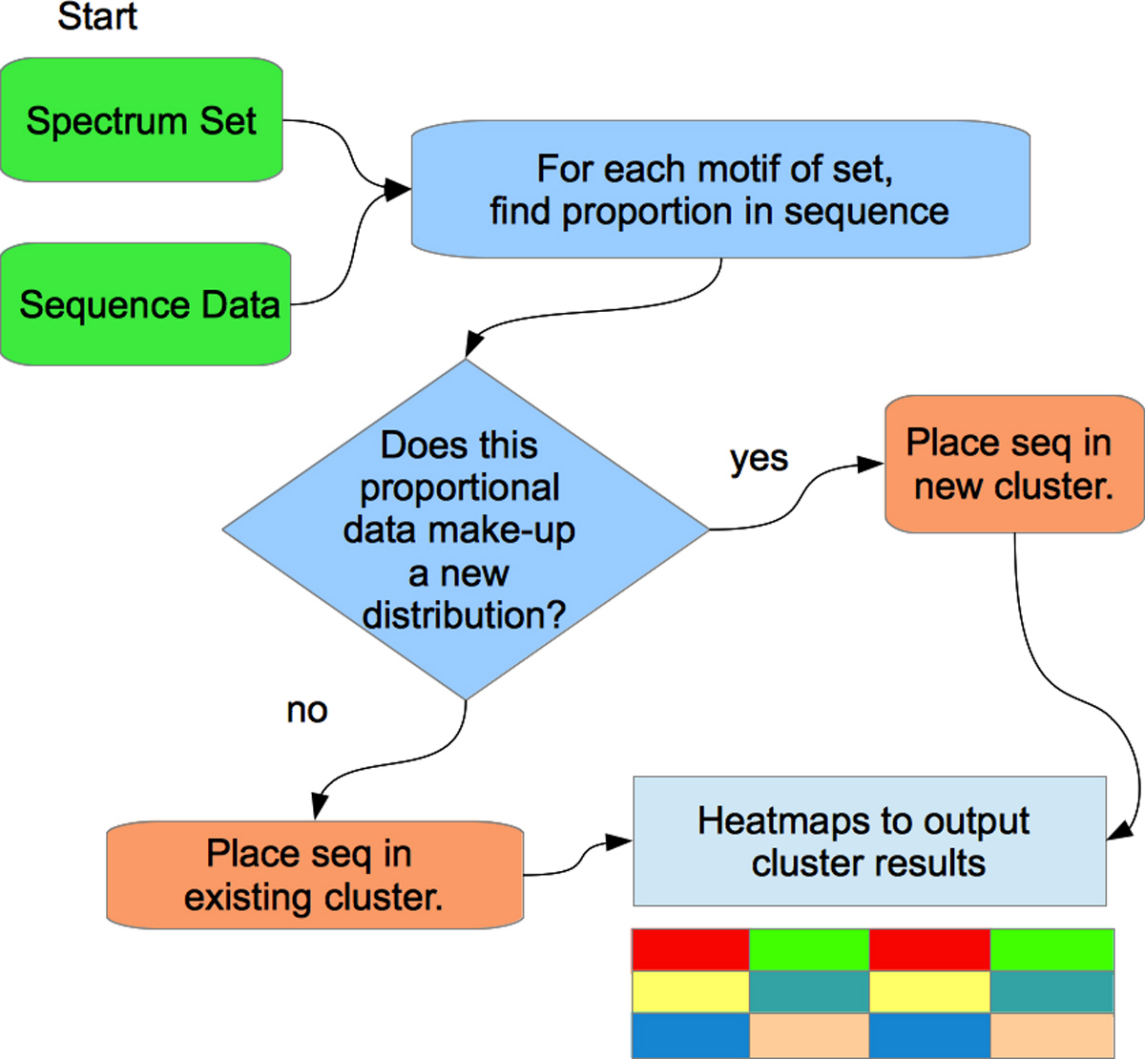
The flowchart that we applied to the clustering operation using heatmaps.

We used the motif proportions, to make vectors from each sample sequence. Comparing the vectors across the organisms determined likenesses and relatedness. If the vectors of the spectrum set motifs were similar between sequences, then this may have been an indication of much common DNA between both sequences. This may also suggest a degree of relatedness between the organisms. Since a contig comes from a sequence, then the contig and the sequence will both share all their DNA and so our analysis will locate these similarity patterns and bin them together. Our analysis code was written in Python and soon will be made available to the bioinformatics community by our software repository located at [23]. In Figure 5, we provide a summary of how our method is applied.

## Results and discussion

According to their proportions of motif content, the clustering in heatmaps describes a tree of relatedness between the organisms. Similar proportions between the sequences are indicated by their close proximity in a *subtree *of the main tree of relatedness. A *parent *sequence is one which is closely related to the sequence from which the reads or contigs were derived. Since these fragments may contain large regions of common code with parent sequence(s), they will associate with them and will be found in its subtree in our heatmaps. By association, we imply that there is ample evidence to suggest that the reads or contigs are more similar to their parent(s) than any other genome in the tree of relatedness. We, furthermore, suggest that these fragments make up a sequence that is related to the parent(s). This property can be utilized to create bins from which to begin assembling each sequence in the reassembly task.

### Sequence data

In the following, we discuss the task of binning long reads or contigs. Here, we choose to use DNA strands of a length 5000 bps. These strands shall be contigs for the purpose of describing the tool that manipulates them. Our method is a tool to determine the proportions of motifs occurring in sequence data. The tool requires enough information from each strand to make correct decisions about relatedness and if there is an insufficient amount of sequence material for comparisons to others, then our base composition tool will make poor determinations. Sequence fragments of 700 bps were often enough to show the trends we discuss in this paper, but we found some errors. We found that longer sequence data provided clearer and more accurate results due to having enough base information upon which our method relies.

This size of sequence material may seem large if the sequences were reads and not contigs. However, we note that sequencing and assembly technologies appear to continually create longer reads than previous technologies. Very large sizes may soon be a reality since read pre-processing methods and various read alignment technologies are already being used to create larger contigs [24-29].

### Clostridium and Staphylococcus

*Clostridium *and *Staphylococcus *typify the kinds of phenomena we observed after of ten trials of each experiment, using the arbitrarily selected pairs of organisms from Table 1. Here will describe the typical kinds of observed phenomena using spectrum sets on these two organisms. We will begin by showing that the two genera groups, *Clostridium *and *Staphylococcus*, are unrelated by the analysis of their motif proportions. We note from Figures 3 and 4 that only *Clostridium*, having the recognition sites *ATTAAT *and *CCCGGG*, can be discriminated by the *AAATTT *and *CCCGGG-*Spectrum sets (Staphylococcus does not have restriction sites of this composition). By our analysis of motif proportions of this spectrum set, we see that both organisms have very different proportions of these spectrum sites.

**Figure 6:**
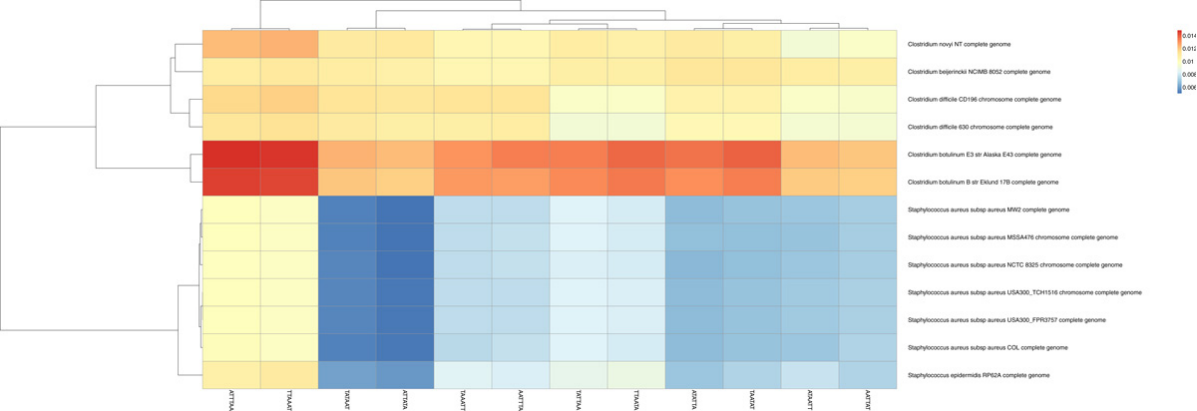
Separation by the *AAATTT*-Spectrum set. There is a clear distinction between each bin; *Closteridium *and *Staphylococcus *of the Firmacute division. The data is segregated except for the two middle sequences forming a separate group. We had similar results from the *AATTCG*-Spectrum set. This result from our previous work in [33].

**Figure 7:**
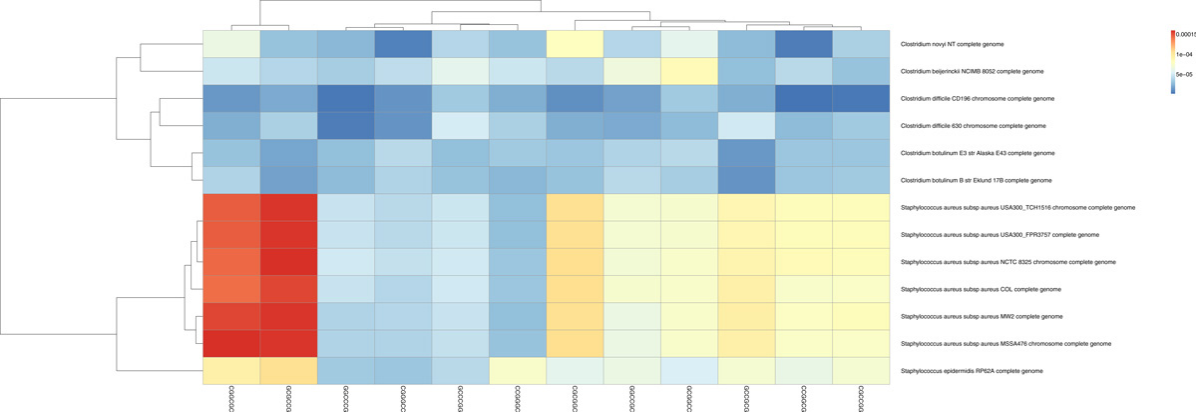
Separation by the motifs of the *CCCGGG*-Spectrum set. Note a clear distinction between each bin. In addition, we note that there is no longer a color pattern showing that *Clostridium botulinum *are closely related, as we saw in Figure 6. This result taken from our previous work in [33].

We note from Figures 6 and 7 that there were two clearly contrasted subtrees in the heatmap to separate the two organisms. There was similar contrast between the sequences of our other heatmaps of the other organisms. In the present two organisms, we noted that the heatmaps are nearly opposite from each other: the Clostridium family members tend to have warmer colors (elevated proportions) and the Staphylococcus members have colder colors (low proportions) in the *AAATTT-*spectrum set. This trend is the inverse for the *CCCGGG-*spectrum set.

The *AATTCG-*spectrum set was also successful in showing two different family subtrees but there was much less apparent contrast between the organisms than there was when using the *AAATTT-*spectrum set. We attribute this high contrast to the phenomenon that a spectrum set may perhaps be more biologically relevant to one of the organisms than the other, according to their recognition sequence usage. The *CCGGAT-*spectrum set was not typically very successful in showing contrasts for binning in our trials for these organisms. This same experiment was performed ten times with different (i.e., newly selected) contigs and we observed similar results in the heatmaps as those discussed. We suggest that since the *Staphylococcus *group appears to have a higher proportion of *CCCGGG *content than *Clostridium*, this contrast helps to associate the reads by relations.

It is clear that the proper use of the correct spectrum set can neatly differentiate one organism group from another for binning. Above, we saw that there are differences in the amounts of the spectrum sets which are found in the organisms. This made a high contrast which helped to determine one organism from another. We will now discuss how this method can discriminate between only read or contig sequence data.

### Proportional differences in contigs by spectrum sets

We shall now discuss an application of separating reads originating from three different organisms that have been mixed together into the same pool. Incidentally, a part of this process comprises the separation of contigs belonging to two different organisms. For our test, we arbitrarily selected another organism (featured in our organism group in Table 1) *Burkholderia pseudomallei *to be added to the contigs from *Clostridium tetani *and *Staphylococcus aureus*. The contigs are of length 5000 bps which we chose to illustrate the test and to showcase its performance.

#### Tests to determine pliable spectrum sets

**Figure 8:**
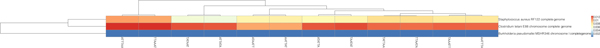
The *AAATTT*-Spectrum set test. The sequence data is applied to our base composition analysis to determine its relatedness.

**Figure 9:**
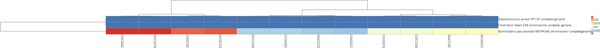
The *CCCGGG*-Spectrum set test. The sequence data is analyzed by base composition to determine relatedness.

**Figure 10:**
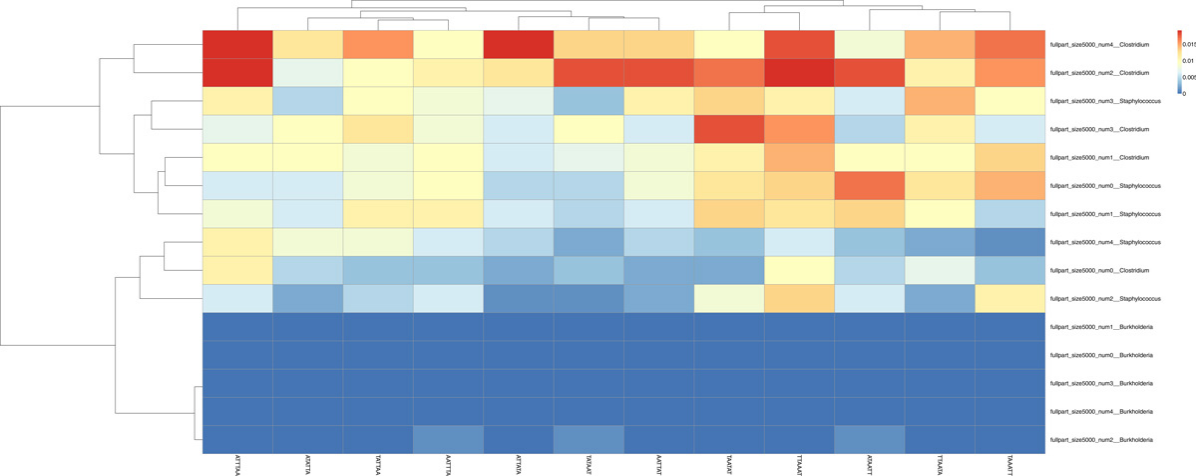
The *AAATTT*-spectrum set analysis taken across all sequence data in a pool. The *Burkholderia pseudomallei *sequence data, having elevated proportions of the motifs of this spectrum set, create a contrast from those of *Clostridium tetani *and *Staphylococcus aureus*. These organisms are observed to have mixed proportions by this heatmap.

**Figure 11:**
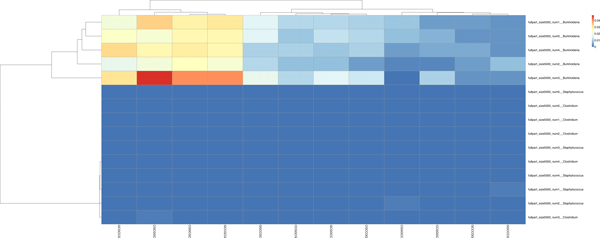
The *CCCGGG-*spectrum set analysis taken across all the contigs in the pool. We note that the *Burkholderia pseudomallei *sequence data, having low proportions of the motifs of this spectrum set, create a contrast from those of *Clostridium tetani *and *Staphylococcus aureus*. These organisms are observed to have mixed proportions by this heatmap.

When working with the contigs of two organisms, a spectrum set could be selected based on the restriction sites which are inherent to the involved organisms. However, a sequencing task may combine contigs of three or more organisms together. The contigs of each organism will have to be separated from those of the other organisms to make the sequence assembly more feasible. Due to the large number of contigs in the whole project, it may not be convenient to run a base composition analysis over all sequence data and so, to determine the spectrum set for the binning task, it is suggested to use the spectrum set test as shown in Figure 8. This test is a base composition analysis taken only across the organisms who are known to be close relatives of the contigs (parents) in the pool. In Figure 8, we note that *Burkholderia *has the lowest proportions of the *AAATTT-*spectrum set. Conversely, in Figure 9, *Staphylococcus *and *Clostridium *have the lowest proportions of the *CCCGGG-*spectrum set. When either of these spectrum sets are applied to the pool of all contigs, we note that the *Burkholderia*, *Staphylococcus *and *Clostridium *contigs reflect the same trends observed at the genome-level. For instance, Figures 10 and 11 reflect the underlining trends of Figures 8 and 9, respectively, in terms spectrum set motif coverage.

#### Removal of the contrasting contig group

**Figure 12:**
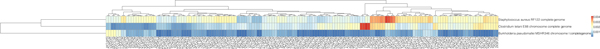
The *AATTCG-*Spectrum set test: The genomes or chromosomes are analyzed by base composition to determine the expected clustering behavior of their contigs.

**Figure 13:**
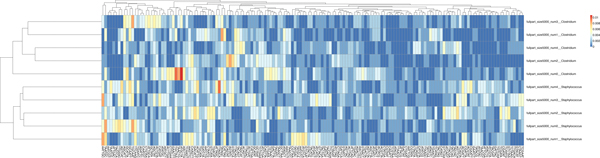
Separation of contigs of *Clostridium tetani *and *Staphylococcus aureus *by the *AATTCG-*spectrum set. We found that this spectrum set worked well to separate the contigs. The *AAATTT-*spectrum set did not perform as well as we had expected from our work in Figure 6. We suggest that the contigs of these particular organisms followed trends shown in Figure 12.

In Figure 8 (spectrum set *AAATTT *), we noted that *Burkholderia *had low proportions of this set, and also in Figure 9 (spectrum set *CCCGGG*, the opposite was true. In Figures 10 and 11, we see that the *Burkholderia *contigs also show this same pattern. Therefore, by this strong contrast, we could remove all contigs which show these strong contrasts and in doing so we would likely be binning the *Burkholderia *contigs. We note that the spectrum set *AATTCG *was unable to to show contrasts between two of three organisms (Figure 12) but *Burkholderia *was still a contrasting group. Interestingly, without this organism, the *AATTCG *spectrum set clearly differentiated *Staphylococcus *and *Clostridium *contigs as shown in Figure 13. This suggests that the addition of *Burkholderia *(having such low proportions of the spectrum set motifs) to the set may change the parameters of the heatmap software.

### Phylogeny from full chromosomes

**Figure 14:**
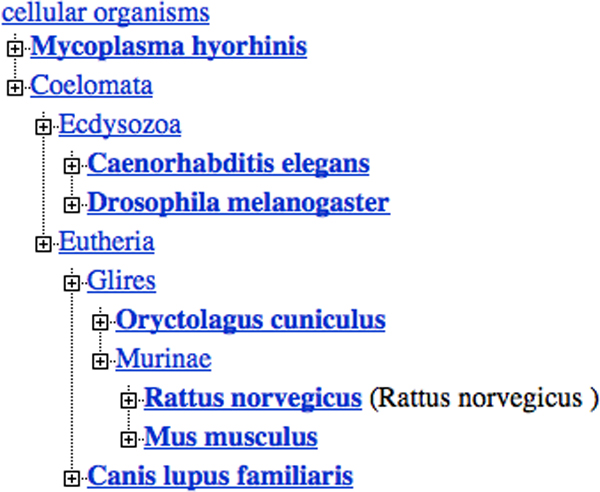
NCBI's Taxonomy Tree used for validation and comparison. This phylogenetic tree was used to compare the results of the spectrum set analysis of the organisms listed in Table 3. We ranked the results on a scale of highest to lowest resemblance in Table 4.

To demonstrate its ability to differentiate sequence data into biologically relevant groups, we show that our method is able to form phylogenetic trees which conform to NCBI's taxonomy tool [30]. In our example, we arbitrarily selected a chromosome from each of seven diverse organisms listed in Table 3. We then applied our framework to extract the distributions of each spectrum set and compared the results to the taxonomy tree in Figure 14 from NCBI which is based on the classification of their taxonomy database and other resources.

**Table 3:** Genomes used in the test

Locus	Organism	Common Name
NC_003279	*Caenorhabditis elegans *chrm1	Worm
NC_006583	*Canis lupus familiaris *chrm 1	Dog
NT_033779	*Drosophila melanogaster *chrm 2L	Fruit fly
NT_039169	*Mus musculus *chrm 1 genomic contig	Mouse
NC_016829	*Mycoplasma hyorhinis *GDL-1 chrm 1	Bacteria
NW_003159226	*Oryctolagus cuniculus *breed Thorbecke inbred chrm1	Rabbit
NW_047544	*Rattus norvegicus *chrm 1	Rat

The organisms used in the base composition experiment. We note that rabbit, dog, mouse and rat are seemingly more closely related than the bacteria, fruit fly and the worm. This observation is used as a *first-glance *assessment of the heatmaps below.

We remind the reader that the subtrees in this example contain organisms that may be related by basic evolutionary phenomena. If we had contigs in the pool from each of these organisms, then these fragments would associate to form more specific family subtrees. Instead, this data is chromosomal sequence material which group by relatedness.

We inspected the resulting trees of this example with the following criteria: the bacterium should be the most evolutionarily distinct organism. The mammals (i.e., the dog, rabbit, rat and mouse) should be the most evolutionarily similar group of the set. The worm and the fruit fly should be found in a subtree which is evolutionarily between the bacterium and the mammals. Indeed, the worm and the fruit fly are quite diverse organisms, however, for this example they are clearly more similar to each other (than to the bacterium) and do not belong to the set of mammals. Therefore, our inspection involved checking for three basic subtrees: one for the mammals, one for the worm and fruit fly, and a subtree containing only the bacterium. In other words, the subtrees had to be arranged similarly to those of NCBI's taxonomy tree shown in Figure 14.

**Figure 15:**
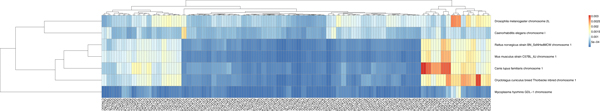
The *CCGGAT-*spectrum set. This tree perfectly resembles the taxonomy tree of Figure 14 and shows the great evolutionary distances between the organisms. The rat and mouse are found to be closely related. We note tree distinct subtrees: one containing the bacterium, one for the mammals and one containing the worm and fruit fly. The location of these subtrees conforms to taxonomy tree.

**Figure 16:**
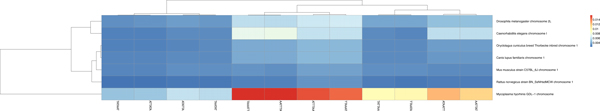
The *AAATTT-*spectrum set. This tree also resembles the taxonomy tree but there is a slight distance between mouse and rat which is not found in Figure 15. We note tree distinct subtrees conforming to the taxonomy tree.

In Figures 15 through 18, we note the phylogenetic trees from each spectrum set. By inspection, the closest trees to the one in Figure 14 are from the *CCGGAT *and *AAATTT *spectrum sets, Figures 15 and 16, respectively. Both of these trees show that the bacterium is most evolutionarily distant from rest of the organisms and that the fruit fly and the worm form a subtree which is distinct from that of the mammals. The locations of the subtrees in both figures are in the same configuration as illustrated in NCBI's taxonomy tree however, the tree of the *AAATTT-*spectrum set is not as accurate as that of the *CCGGAT-*set due to the displayed shorter evolutionary distances (for instance, longer branch lengths indicate more distance). In addition, the distance between rat and mouse is expectedly closer by the *CCGGAT-*spectrum set than by the *AAATTT-*set.

**Figure 17:**
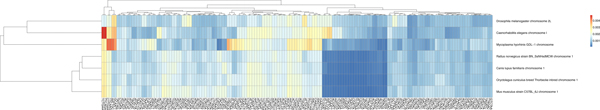
The *AATTCG-*spectrum set. We note that mouse and rat are not closely related. The bacterium is also evolutionarily located between the mammals and the subtree containing the worm and fruit fly.

**Figure 18:**
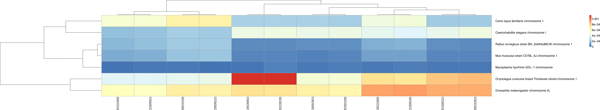
he *CCCGGG-*spectrum set. This tree is inaccurate because it indicates that the rabbit and the fruit fly are closely related.

The tree from the *AATTCG-*spectrum set in Figure 17 shows that the bacterium is evolutionarily found between the mammal's subtree and that of the worm and fruit fly. This is inaccurate by the taxonomy tree Figure 14. In addition, the tree from the *CCCGGG-*spectrum set (Figure 18) is also inaccurate since it shows that the fruit fly is closely related to the rabbit. These results confirm our earlier findings that the choice of the correct spectrum set is of paramount importance for a successful analysis.

**Figure 19:**
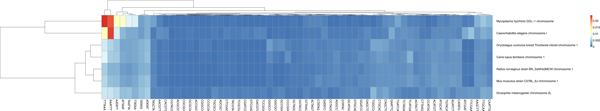
Length-6 palindromic spectrum set. Here we note that this tree does not conform well to the validation tree in Figure 14. Rat and mouse are shown to be closely related but inaccurately, the tree shows that the bacterium and the worm are also closely related.

Since the spectrum set motifs were originally inspired from palindromic restriction sites, we also studied the proportions of an exhaustive list of length-6 palindromic motifs (64 in total) across the sequence data. Interestingly, although palindromic motifs have been known to successfully differentiate chromosomes, as shown in [14]. However in Figure 19, we note that palindromes do not successfully recreate the taxonomy tree from Figure 14.

To summarize these results, we offer Table 4 which contains the highest to lowest resemblance to the tree in Figure 14. We note from their ranking that the spectrum sets do not behave uniformly and that further study is required to understand how they should be applied to a particular set of organismal data for classification.

**Table 4:** Ranking of sets

Ranking	Set Seed	Figure
1	CCGGAT	15
2	AAATTT	16
3	AATTCG	17
4	Palindromes, Length-6	19
5	CCCGGG	18

Ranking of Spectrum Sets over Chromosomal Data: We note the best to worse resemblance of spectrum set trees to actual taxonomy data. For this data set, the *CCGGAT-*spectrum set created a tree which most closely resembled the one based on the classification in NCBI taxonomy database in Figure 14.

## Conclusion

As in playing with jigsaw puzzles, if there are the pieces of several different puzzles in the same box, then the completion of any one of the puzzles is a sizable undertaking. In the same way, during a sequence assembly task where the contigs of different organisms are mixed together in the pool, much time can be spared by first sorting the contigs into their own bins from which to work. Our method places many of the unknown contigs into their corresponding bins to drastically reduce the search space.

Most of this paper discussed working with contigs which are typically longer than reads. Our base composition analysis tool works by quantifying the amount the spectrum set motifs which are contained in the sequence data. When there is not enough sequence data, then our method may produce erroneous results and so we suggest using contigs of at least 1000 bps because they should contain enough sequence information for a good analysis. We should mention here that we have had very good results when using contigs of 700 bps in length and so 1000 bps is not always absolutely necessary.

By sorting the contigs with related sequence data which is based on motif proportions, our method aims to accomplish the binning task. We used heatmaps to show the contig clusters by organism-types. Furthermore, we illustrated that there are only four spectrum sets, which can be created from length-6 recognition sites to apply to differentiate by contrasting the sequence data. For instance, we used the *AAATTT *and *CCCGGG-*spectrum sets to show that one set had high proportional values in one organism, but not the other. This created the contrast that would help to bin the contigs of these two organisms. We then showed how the contigs of three organisms can be binned in a two-step process. We first removed the most contrasting set of contigs in the pool and then reapply our method to the remaining contigs. An analysis by base composition can also be used to determine evolutionary orders of organismal sequence data. For instance, we showed that our method could create phylogenetics trees which were very similar to those produced by NCBI's taxonomy tool.

One of the leading benefits to our method is that there is no setup required as there would be for other sequence recognition softwares such as BLAST [31] or BLAT [32]. While these methods provide powerful sequence analysis, they require expansive hardware requirements for use (i.e., memory, storage and fast computational power). Our method is a statistical approach, programmed in Python to run on basic hardware and does not require a database for operation.

Our goals for the future are to test this base composition framework using synthetic and biological data to further analyze its performance and levels of sensitivity. This work will be conducted using MetaSim, to generate contigs of a 10 X coverage for two or more genomes which we shall apply to our binning method. This study will help to give us a more realistic interpretation of its power for discriminating contigs and how best to use it as a pre-processing step to sequence assembly.

## Competing interests

The authors declare that they have no competing interests.

## Authors' contributions

OBC and DB developed the idea, realized the program, tested the software and drafted the manuscript. HA helped to shape the features of the application. All authors read and approved the manuscript.

## Declarations

The corresponding authors have provided the funding for publication of the article.

This article has been published as part of *BMC Bioinformatics *Volume 14 Supplement 11, 2013: Selected articles from The Second Workshop on Data Mining of Next-Generation Sequencing in conjunction with the 2012 IEEE International Conference on Bioinformatics and Biomedicine. The full contents of the supplement are available online at http://www.biomedcentral.com/bmcbioinformatics/supplements/14/S11.

## References

[B1] Kunin V Copeland A Lapidus A Mavromatis K Hugenholtz P A bioinformatician's guide to metagenomics Microbiology and molecular biology reviews: MMBR 2008 72 4 557 578 10.1128/MMBR.00009-08 19052320 PMC2593568

[B2] Wu YW Ye Y Berger B, Berlin, Heidelberg A Novel Abundance-Based Algorithm for Binning Metagenomic Sequences Using l-Tuples Research in Computational Molecular Biology, Volume 6044 of Lecture Notes in Computer Science 2010 Springer Berlin/Heidelberg 535 549

[B3] Kembel SW Eisen JA Pollard KS Green JL The Phylogenetic Diversity of Metagenomes PLoS ONE 2011 6 8 e23214+ http://dx.doi.org/10.1371/journal.pone.0023214 21912589 10.1371/journal.pone.0023214 PMC3166145

[B4] Schuster S Next-generation sequencing transforms today's biology Nature Methods 2008 5 16 18 18165802 10.1038/nmeth1156

[B5] Li R Zhu H Ruan J Qian W Fang X Shi Z Li Y Li S Shan G Kristiansen K Li S Yang H Wang J Wang J De novo assembly of human genomes with massively parallel short read sequencing Genome Res 2010 20 265 272 10.1101/gr.097261.109 20019144 PMC2813482

[B6] Hossain M Azimi N Skiena S Crystallizing short-read assemblies around seeds BMC Bioinformatics 2009 10 Suppl 1 S16 10.1186/1471-2105-10-S1-S16 19208115 PMC2648751

[B7] Bryant DW Jr Wong WK Mockler TC QSRA - a quality-value guided de novo short read assembler BMC Bioinformatics 2009 10 69 10.1186/1471-2105-10-69 19239711 PMC2653489

[B8] Wang Y Zeng X Iyer NJ Bryant DW Mockler TC Mahalingam R Exploring the switchgrass transcriptome using second-generation sequencing technology PloS One 2012 7 3 e34225 10.1371/journal.pone.0034225 22479570 PMC3315583

[B9] Garg R Patel R Tyagi A Jain M De Novo Assembly of Chickpea Transcriptome Using Short Reads for Gene Discovery and Marker Identification DNA Research 2011 18 53 63 10.1093/dnares/dsq028 21217129 PMC3041503

[B10] Ji Y Shi Y Ding G Li Y A new strategy for better genome assembly from very short reads BMC Bioinformatics 2011 12 493 10.1186/1471-2105-12-493 22208765 PMC3268122

[B11] Zerbino D McEwen G Margulies E Birney E Pebble and rock band: heuristic resolution of repeats and scaffolding in the velvet short-read de novo assembler PLoS One 2009 4 e8407 10.1371/journal.pone.0008407 20027311 PMC2793427

[B12] Zhang W Chen J Yang Y Tang Y Shang J Shen B A practical comparison of de novo genome assembly software tools for next-generation sequencing technologies PLoS One 2011 6 e17915 10.1371/journal.pone.0017915 21423806 PMC3056720

[B13] Peng Q Smith A Multiple sequence assembly from reads alignable to a common reference genome IEEE/ACM Trans Comput Biol Bioinform 2011 8 1283 1295 21778524 10.1109/TCBB.2010.107

[B14] Lamprea-Burgunder EMP Ludin P Species-specific Typing of DNA Based on Palindrome Frequency Patterns DNA Res 2011 18 2 117 24 10.1093/dnares/dsr004 21429991 PMC3077040

[B15] Hershberg R Petrov D Evidence that mutation is universally biased towards AT in bacteria PLoS Genet 2010 6 e1001115 10.1371/journal.pgen.1001115 20838599 PMC2936535

[B16] Hildebrand F Meyer A Eyre-Walker A Evidence of selection upon genomic GC-content in bacteria PLoS Genet 2010 6 e1001107 10.1371/journal.pgen.1001107 20838593 PMC2936529

[B17] Lightfield J Fram N Ely B Across bacterial phyla, distantly-related genomes with similar genomic GC content have similar patterns of amino acid usage PLoS One 2011 6 e17677 10.1371/journal.pone.0017677 21423704 PMC3053387

[B18] Richter D Ott F Auch A Schmid R Huson D MetaSim: a sequencing simulator for genomics and metagenomics PLoS One 2008 3 e3373 10.1371/journal.pone.0003373 18841204 PMC2556396

[B19] Roberts R Vincze T Posfai J Macelis D REBASE--a database for DNA restriction and modification: enzymes, genes and genomes Nucleic Acids Res 2010 38 D234 D236 10.1093/nar/gkp874 19846593 PMC2808884

[B20] Gelfand M Koonin E Evidence of selection upon genomic GC-content in bacteria Avoidance of palindromic words in bacterial and archaeal genomes: a close connection with restriction enzymes 1997 25 12 2430 2439 10.1093/nar/25.12.2430 PMC1995031 9171096

[B21] R Development Core Team R: A language and environment for statistical computing R Foundation for Statistical Computing 2012

[B22] Kolde R pheatmap: Pretty Heatmaps. R package version 0.6.1 2012 http://CRAN.R-project.org/package=pheatmap

[B23] http://bioinformatics.ist.unomaha.edu/softwares.php?p=softwares

[B24] Edgar RC MUSCLE: multiple sequence alignment with high accuracy and high throughput Nucleic acids research 2004 32 5 1792 1797 http://dx.doi.org/10.1093/nar/gkh340 10.1093/nar/gkh340 15034147 PMC390337

[B25] Zerbino DR Birney E Velvet: Algorithms for de novo short read assembly using de Bruijn graphs Genome Research 2008 18 5 821 829 http://dx.doi.org/10.1101/gr.074492.107 10.1101/gr.074492.107 18349386 PMC2336801

[B26] Simpson JT Wong K Jackman SD Schein JE Jones SJM Birol I ABySS: A parallel assembler for short read sequence data Genome Research 2009 19 6 1117 1123 http://dx.doi.org/10.1101/gr.089532.108 10.1101/gr.089532.108 19251739 PMC2694472

[B27] Li R Zhu H Ruan J Qian W Fang X Shi Z Li Y Li S Shan G Kristiansen K Li S Yang H Wang J Wang J De novo assembly of human genomes with massively parallel short read sequencing Genome Research 2010 20 2 265 272 http://dx.doi.org/10.1101/gr.097261.109 10.1101/gr.097261.109 20019144 PMC2813482

[B28] Schmutz J Cannon SB Schlueter J Ma J Mitros T Nelson W Hyten DL Song Q Thelen JJ Cheng J Xu D Hellsten U May GD Yu Y Sakurai T Umezawa T Bhattacharyya MK Sandhu D Valliyodan B Lindquist E Peto M Grant D Shu S Goodstein D Barry K Futrell-Griggs M Abernathy B Du J Tian Z Zhu L Gill N Joshi T Libault M Sethuraman A Zhang XC Shinozaki K Nguyen HT Wing RA Cregan P Specht J Grimwood J Rokhsar D Stacey G Shoemaker RC Jackson SA Genome sequence of the palaeopolyploid soybean Nature 2010 463 7278 178 183 http://dx.doi.org/10.1038/nature08670 10.1038/nature08670 20075913

[B29] Paszkiewicz K Studholme DJ De novo assembly of short sequence reads Briefings in Bioinformatics 2010 11 457 472 10.1093/bib/bbq020 20724458

[B30] http://www.ncbi.nlm.nih.gov/Taxonomy/CommonTree/wwwcmt.cgi

[B31] Altschul SF Madden TL Schäffer AA Zhang J Zhang Z Miller W Lipman DJ Gapped BLAST and PSI-BLAST: a new generation of protein database search programs Nucleic Acids Res 1997 25 3389 3402 10.1093/nar/25.17.3389 9254694 PMC146917

[B32] Kent WJ BLAT--The BLAST-Like Alignment Tool Genome Research 2002 12 4 656 664 11932250 10.1101/gr.229202 PMC187518

[B33] Bonham-Carter O Ali H Bastola D A meta-genome sequencing and assembly preprocessing algorithm inspired by restriction site base composition Bioinformatics and Biomedicine Workshops (BIBMW), 2012 IEEE International Conference: 4-7 October 2012 2012 696 703 10.1109/BIBMW.2012.6470222

